# Immediate Feedback Improves Career Decision Self-Efficacy and Aspirational Alignment

**DOI:** 10.3389/fpsyg.2019.00255

**Published:** 2019-02-13

**Authors:** Nathan Berger, Jose Hanham, Catherine J. Stevens, Kathryn Holmes

**Affiliations:** ^1^Centre for Educational Research, Western Sydney University, Kingswood, NSW, Australia; ^2^MARCS Institute for Brain, Behaviour and Development, and School of Social Sciences and Psychology, Western Sydney University, Bankstown, NSW, Australia

**Keywords:** career aspirations, misalignment, self-efficacy, intervention studies, feedback

## Abstract

Misalignment between career and education aspirations has been associated with poorer achievement during adolescence and unstable employment in adulthood. In this study, we evaluated whether a brief in-school intervention improved career decision self-efficacy and aspirational alignment. We sampled 211 teenagers living in disadvantaged areas of Western Sydney, Australia using a quasi-experimental non-equivalent groups design. Students completed pre- and post-questionnaires which measured aspirational alignment and career decision self-efficacy. Students in the intervention condition (*n* = 102) received automated feedback on the alignment of their career and education aspirations, as well as a career information pamphlet detailing the educational pathways to a range of popular careers. Students in the control condition completed both questionnaires but only received feedback and the pamphlet at the end of the study. The intervention improved alignment of career and education aspirations, as well as increased some dimensions of career decision self-efficacy. Students in the intervention group more frequently identified the correct qualification for their career aspiration in the post-questionnaire (57.9%) compared with the pre-questionnaire (48.1%). Students with misaligned aspirations in the intervention group reported higher self-efficacy for gathering occupational information and selecting goals following the intervention. There were no pre-post differences for students in the control condition. The practical significance of this study is that an easy, low-cost intervention can improve aspirational alignment between career and education aspirations, as well as aspects of career decision self-efficacy.

## Introduction

Misalignment between career and educational aspirations occurs when the minimum qualification required for a person’s desired occupation exceeds their educational expectations (Perry et al., 2016). Poor alignment is associated with volatile aspiration trajectories during schooling (Berger, 2016), lower achievement at the conclusion of secondary school (Schmitt-Wilson and Faas, 2016), lower university entry rates (Morgan et al., 2013), and unstable employment and lower wage attainment in adulthood (Sabates et al., 2011). Students from disadvantaged backgrounds appear more prone to aspirational misalignment, despite generally not having ‘lower’ career aspirations compared to their more advantaged peers (Gore et al., 2015; Perry et al., 2016). Disadvantaged young people often have less exposure to higher education and professional occupations, increasing their susceptibility to aspirational misalignment and perpetuating socioeconomic inequalities (Perry et al., 2016).

A fundamental aim of careers education is to teach students to make informed academic and occupational decisions (Uffelman et al., 2004). However, this task is complicated by modern social and economic settings which have increased occupational mobility and require individuals to make more frequent job transitions during their lifetimes (Savickas, 2012; Santisi et al., 2018). While building students’ knowledge through identification of a career and qualification of interest historically has been a staple activity of careers education (Savickas, 2012), modern adolescents also need more transferable psychological resources to enable navigation of the less linear career pathways they are likely to experience in late modernity (Formica et al., 2017; Santisi et al., 2018).

Career decision self-efficacy (CDSE) focuses on the capacities needed to successfully navigate academic and occupational pathways, such as gathering occupational information, selecting goals, planning pathways, solving problems, and self-appraisal of interests and abilities (Betz et al., 1996). Higher levels of CDSE are associated with lower levels of career indecision, openness to a greater range of occupations, better career adjustment, and rational career-decision making patterns (Uffelman et al., 2004; Brown and Lent, 2016). However, there are mixed findings concerning whether demographic factors like gender, race/ethnicity and socioeconomic background are associated with CDSE (Choi et al., 2012). Some studies found a strong association between CDSE and these factors (Creed et al., 2002; Metheny and McWhirter, 2013), whilst others found no association (Gushue and Whitson, 2006), leading some scholars to conclude that the association is likely indirect, mediated or moderated by learning experiences (Choi et al., 2012).

Relatively brief career education interventions have proven successful in motivating academic choices and improving career-related self-efficacy in school students (Turner and Lapan, 2005; Harackiewicz et al., 2012; Rozek et al., 2015). Harackiewicz et al. (2012) conducted a field experiment testing whether brochures mailed to parents could increase participation in senior school science and mathematics, by emphasizing the utility-value of these subjects. Students in the intervention group took on average an extra semester more of these subjects in the final years of high school, compared to students in the control group. The intervention was most effective with low achieving boys and high achieving girls (Rozek et al., 2015). Turner and Lapan (2005) conducted a quasi-experimental study wherein adolescents gave their perceptions about a variety of careers and received personalized computer-generated recommendations for further exploration of career and education information. For students in the experimental condition, the hour-long intervention increased their career-related self-efficacy and interest in gender non-traditional careers.

We tested a brief intervention designed to improve adolescents’ aspirational alignment and CDSE. High school students were given immediate, personalized feedback in an online survey of their career and education aspirations, and received a careers information pamphlet. We had two hypotheses:

H1. A higher proportion of students who receive automated personalized feedback (intervention) will be aligned compared to students who do not receive such feedback (control) after three weeks.
H2. Students who receive automated personalized feedback (intervention) will have higher mean scores on CDSE subscales after three weeks while students who do not receive such feedback (control) will not.

## Materials and Methods

### Ethics Approval and Data Availability

This study was carried out in accordance with the National Statement on Ethical Conduct in Human Research, National Health and Medical Research Council (Australia). The protocol was approved by the Human Research Ethics Committee at Western Sydney University. All participants and their parents/guardians gave written informed consent in accordance with the Declaration of Helsinki. The datasets for this manuscript are not publicly available because participants did not consent to this. Requests to access the datasets should be directed to Dr. Nathan Berger, n.berger@westernsydney.edu.au

### Participants

Participants were students aged 14–16 years (*n =* 211, *M =* 15.05, *SD* = 0.77) conveniently sampled from two systemic Catholic high schools in Western Sydney, Australia. All students (*n =* 369) undertaking careers education at the schools were invited to voluntarily participate in November 2017. The response rate was 57%.

### Procedure

A quasi-experimental non-equivalent groups design was used to randomly assign entire classes of participants to intervention and control conditions. This design responds to concerns that it is unethical and impracticable to differentially intervene with some children but not others within the same classroom (Handley et al., 2011). In non-equivalent designs, intact groups with similar characteristics are assigned randomly to each condition (Turner and Lapan, 2005). Although this design is not as strong as randomly assigning individuals, it is useful in schools because intact classes are already formed (Turner and Lapan, 2005). A total of 102 students (48%) were assigned to the intervention condition.

All participants completed online pre- and post-questionnaires. The intervention condition questionnaire exclusively contained a feedback mechanism (described below). Three weeks elapsed between sampling occasions. In the second week, intervention participants were mailed a careers information pamphlet, detailing teenagers’ top 10 career aspirations (see Gore et al., 2015) and the corresponding qualifications. Control participants only received this pamphlet at the study’s conclusion.

#### Instruments

The pre-questionnaire gathered students’ age, gender, postcode, language background, and parents’ occupation and qualification. Students’ CDSE was measured using the previously-validated CDSE Scale Short Form (see Betz et al., 1996 for psychometric characteristics). Students used a five-point Likert scale to report their confidence at tasks under the five CDSE subscales of occupational information (OI), goal selection (GS), planning pathways (PP), problem solving (PS), and self-appraisal (SA). Students were also asked what occupation and qualification they aspired to attain by age 25 (see Gore et al., 2015) by selecting from the full-list of 919 occupations and 5 qualification levels in the *Australian and New Zealand Standard Classification of Occupations* (ANZSCO; Australian Bureau of Statistics, 2006). Aspirational alignment was calculated as ‘aligned’ or ‘misaligned’ depending on whether the correct qualification was selected for the occupation as defined by ANZSCO. The post-questionnaire only collected the CDSE and aspirations information.

The intervention condition questionnaires provided immediate feedback about whether the correct qualification had been identified for the career and, if not, what the correct qualification would be. Following this feedback, intervention participants were asked to match qualifications to the top 14 career aspirations reported by Australian teenagers (see Gore et al., 2015). The career they had nominated was automatically added at a random position, serving to immediately check feedback efficacy. Control participants only received this feedback at the conclusion of the post-questionnaire.

## Results

Only data from participants who completed both pre- and post-questionnaires are reported here. There was a low level of missing data associated with student attendance at sampling occasions. Alpha reliability coefficients for the CDSE subscales were acceptable (0.72 < α < 0.82). All inferential tests are reported at a 95% confidence level. Data were analyzed using IBM SPSS Statistics Version 25.

### Sample Characteristics

Due to the non-equivalent groups design preserving existing class characteristics, the intervention condition had slightly more males (55, 53.9%) than females (47, 46.1%), while the control condition had slightly fewer males (53, 48.6%) than females (56, 51.4%). Students in both conditions were aged between 14 and 16, with a mean age of 15 years. As the schools were located in the culturally and linguistically diverse Western Sydney region, nearly half of all students in both conditions (103, 48.8%) spoke a language other than English at home, most frequently Arabic, Hindi, and Tagalog (Filipino). In terms of parental education as reported by students, 88 (23%) had a bachelor’s degree or higher, 51 (13%) had a high school qualification, 32 (8%) had a vocational qualification, while 218 (56%) had an education that was ‘unknown’ to students. After discussions with the schools, we surmise that a contributing factor to this figure was migrant parents having an education which was not easily converted into an Australian equivalent by students, or in some cases, refugee parents having had little or no formal education in their country of origin.

### Aspirational Alignment

McNemar’s exact test of difference compared the proportions of students whose expectations were aligned/misaligned at the different sampling points (Table 1). There was a significant increase in the proportion of aligned students immediately after feedback. After three weeks, the aligned proportion was still higher than baseline but not significantly so. Another round of feedback again significantly increased the proportion of aligned students compared to baseline, and to a higher percentage than the first round of feedback. In the control condition, there was no significant differences after three weeks compared to baseline. Once students in the control condition received the end-of-study feedback, they demonstrated alignment at a similar proportion to the intervention condition after their first round of feedback. The same patterns held when we tested separately by gender, language background, and parental education.

**Table 1:** Test of difference for aligned and misaligned students within quasi-experimental groups.

	Pre-, *n* (%)		Post-, *n* (%)	
		Feedback	Delayed	Feedback
	Baseline	check	re-test	check
*Intervention*				
Aligned	49 (48.0%)	75 (78.9%)^∗^	58 (56.9%)	74 (88.2%)^∗^
Misaligned	53 (52.0%)	20 (21.1%)^∗^	44 (43.1%)	16 (17.8%)^∗^
*Control*				
Aligned	56 (51.4%)	No feedback	63 (57.8%)	78 (77.2%)^∗^
Misaligned	53 (48.6%)	No feedback	46 (42.2%)	23 (22.8%)^∗^


*^∗^Significantly different to baseline, McNemar exact *p <* 0.05.*

H1 was supported with a caveat. Exposure to personally-relevant careers information improved students’ ability to correctly identify the qualification of their career aspiration. However, repeated feedback appears necessary to sustain the improvement over time.

### CDSE

Paired-samples *t*-tests compared CDSE scales between sampling occasions within quasi-experimental groups (Table 2). Intervention students showed significant increases in OI, GS, and Total CDSE. We also tested separately by gender, language background, and parental education. Post-intervention, females were higher on GS (3.56–3.83, *t*(46) = -2.82, *p* = 0.007), PS (3.57–3.85, *t*(46) = -2.89, *p* = 0.006), and Total CDSE (3.62–3.81, *t*(44) = -2.23, *p* = 0.031); students from non-English-speaking backgrounds were higher on GS (3.61–3.88, *t*(43) = -2.59, *p* = 0.013); and students with high school-educated parents were higher on OI (3.45–3.82, *t*(10) = -2.56, *p* = 0.029). There were no significant differences in any of these factors in the control condition.

**Table 2:** Paired *t*-tests for CDSE between sampling occasions within quasi-experimental groups.

			Paired *t*-test				
	Pre-, *M* (*SD*)	Post-, *M* (*SD*)	*t*	*df*	*p*	*d*
*Intervention*						
OI	3.67 (.66)	3.80 (.64)	-2.02	100	0.046*	0.21
GS	3.63 (.63)	3.79 (.61)	-2.59	101	0.011*	0.27
PP	3.68 (.71)	3.74 (.66)	-0.94	99	0.343	0.10
PS	3.65 (.67)	3.72 (.62)	-1.18	100	0.243	0.12
SA	3.82 (.68)	3.91 (.56)	-1.67	100	0.099	0.16
Total CDSE	3.68 (.60)	3.78 (.54)	-1.99	98	0.049*	0.20
*Control*						
OI	3.64 (.72)	3.69 (.69)	-0.78	107	0.440	0.08
GS	3.61 (.66)	3.66 (.62)	-0.95	107	0.345	0.09
PP	3.62 (.78)	3.64 (.71)	-0.30	106	0.766	0.03
PS	3.61 (.69)	3.68 (.66)	-1.16	107	0.247	0.12
SA	3.79 (.79)	3.83 (.66)	-0.67	107	0.505	0.07
Total CDSE	3.64 (.66)	3.69 (.59)	-1.01	105	0.315	0.11


*^∗^*p <* 0.05, unadjusted for multiple comparisons (Freise, 2002)*

H2 was partially supported. Exposure to personally-relevant careers information improved aspects of students’ CDSE. The intervention only improved efficacy in gathering occupational-information and selecting goals, but this was sufficient to improve overall career decision self-efficacy.

## Discussion

Misalignment between career and education aspirations in adolescence has been associated with several negative outcomes in adulthood (Perry et al., 2016). Adolescents also need transferable psychological resources to enable successful navigation of the less linear career pathways they are likely to experience in modern times (Santisi et al., 2018). We investigated whether a brief in-school intervention could improve teenagers’ alignment capability and CDSE. We found that exposure to personally-relevant careers information improved alignment capability over the short term. In the intervention condition, a higher proportion of students were aligned immediately after receiving feedback, regardless of their gender, language background or parental education. However, repeated exposure is likely required to sustain this improvement over a longer period of time. A question arising from this study is: when and how often should students be exposed to the protocol in order for educational and career alignment to be sustained over the long-term?

As in previous studies (Creed et al., 2002; Gushue and Whitson, 2006; Choi et al., 2012), we found that gender, language background (an imperfect proxy for cultural/ethnic background), and parental education (a proxy for socioeconomic status) were related to some CDSE subscales, but not others. In the intervention condition, girls experienced positive shifts in their goal selection and problem-solving; students from non-English-speaking backgrounds saw improved goal selection; and students with parents whose highest education was secondary school had better occupational information scores after the intervention. The effect sizes for these improvements ranged from 0.20–0.27, which is consistent with previous intervention research on career self-efficacy (Turner and Lapan, 2005). Overall, the occupational information and goal selection subscales appeared to be more malleable by the intervention as presently designed, given its focus on identifying and giving feedback on the match between specific career and education aspirations.

## Limitations and Future Research

There are several limitations which affect the generalisability of this study. The quasi-experimental design which preserved intact class groups was a pragmatic choice in the Australian research ethics context, but is less methodologically rigorous than a pure experimental design. It is possible that the results were influenced by external agents, such as teachers and peers, because these cannot (and arguably, should not) be controlled in the naturalistic setting of the classroom. The study also had a relatively small sample size drawn from a limited range of participants in terms of age and geography. Future studies could investigate the brief intervention described here with a wider range of participants in full experimental designs where the researchers can be more certain about the absence of contamination effects. The inclusion of a post-intervention follow-up could also verify the stability of the results.

Given contemporary social and economic settings have increased occupational mobility (Santisi et al., 2018), future studies might also treat alignment as a general capability and examine students’ abilities across a range of career and education pathways including alternate pathways not captured by the one-to-one correspondence between careers and education defined by ANZSCO. Such a generalized capability might be better related to PP, PS, and SA, and might moderate CDSE scores between pre- and post-sampling occasions. While we were interested in a brief and relatively simple intervention, future studies might consider more articulated interventions that examine the various dimensions proposed by the ‘life design’ paradigm (see Savickas, 2012).

## Conclusion

Despite these limitations, this study makes a significant contribution to the career education literature. It further highlights the promise of relatively brief interventions established by previous studies (e.g., Turner and Lapan, 2005; Harackiewicz et al., 2012) but also represents an innovation through the use of computerized but personalized feedback as part of a brief intervention. Such automated feedback on students’ career and education aspirations can improve aspirational alignment and career decision self-efficacy and represents a promising avenue for future research and intervention work in schools.

## Author Contributions

NB, JH, and KH developed the research from an initial idea by NB and KH. NB and JH collected and analyzed the data. NB wrote the first draft of the paper. All authors helped to finalize the paper.

## Conflict of Interest Statement

The authors declare that the research was conducted in the absence of any commercial or financial relationships that could be construed as a potential conflict of interest.
